# Impact of named caps on anesthesia and intensive care team performance: a randomized simulation study

**DOI:** 10.1016/j.bjane.2026.844812

**Published:** 2026-09-04

**Authors:** Sylvain Garnier, Laurent Mattatia, Nicolas Boulet, Gregoire Bosselut, Chris Serrand, Sylvain Benenati, Philippe Cuvillon, Laurent Muller, Lionel Brunel, Claire Roger

**Affiliations:** a UR-UM103 IMAGINE, Univ Montpellier, Division of Anesthesia and Critical Care, Pain and Emergency Medicine, Nîmes University Hospital, Montpellier, France; b Nîmes Universitary Hospital Simulation Center (SimHU), Nîmes, France; c Department of Psychology, Epsylon Laboratory, University Paul Valéry, Montpellier, France; d EuroMov Digital Health in Motion, Univ Montpellier, IMT Mines Ales, France; e Biostatistic, Epidemiology, Public Health and Methodology Department, Nîmes University Hospital, Nîmes, France

**Keywords:** Anesthesiology, Clinical Competence, Communication, Patient care team, Patient safety, Simulation training

## Abstract

**Background:**

Effective communication and team non-technical skills are key points for patient safety in anesthesia and intensive care. Named caps have been proposed as a strategy to enhance team efficiency. However, evidence regarding their impact on objectively assessed team performance remains limited.

**Methods:**

We conducted a prospective, single center, randomized, and experimental study in a high-fidelity simulation setting (SimHU, Nîmes). Anesthesia and intensive care residents, young doctors, and paramedics participated in simulated critical scenarios, alternating between sessions with and without named caps. The primary objective was to compare team performance, assessed by observers using the TEAM score. The secondary objectives were self-reported measures of entitativity, team cohesion and inclusion of self in the group.

**Results:**

A total of 112 participants across 32 simulation sessions were included, which were assessed by 19 instructors. TEAM scores were significantly higher in the named cap group compared to the control group: the 44 points TEAM score (37 ± 7 vs. 33 ± 7; p = 0.026) and the total score (45 ± 8 vs. 40 ± 9; p = 0.020). Subjective perceptions of entitativity and team cohesion did not differ significantly.

**Conclusions:**

Named caps were associated with higher team performance in a simulated environment. Their impact on perceived team cohesion and entitativity was not statistically significant under simulation conditions. Further research in clinical settings is needed to determine whether named caps improve teamwork and patient safety.

## Introduction

Teams working in anesthesia and intensive care units are complex interprofessional systems composed of individuals with diverse professions, personalities, and skills, all working toward the shared goal of managing critically ill patients through effective collaboration. Directed communication is a major determinant of patient safety during perioperative and critical care management.^1^ In the operating room, structured intraoperative briefing involving surgeons, anesthetists and nurses has been associated with reductions in mortality, unplanned reoperations, and hospital length of stay.^2^ Beyond interprofessional communication, rapid and accurate identification of team members may also contribute to quality of care. Facilitated identification may promote task coordination and communication among professionals,^3^ thereby enhancing teamwork and collective adaptive capacity. Wearing named caps represents a simple and safe strategy to facilitate identification, particularly in operating rooms where conventional identification badges are often concealed by sterile gowns and personal protective equipment. Named caps may therefore support teamwork and improve adaptive team functioning.^4^^,^^5^

According to the teamwork model proposed by Gregory et al.,^6^ team adaptation depends on the implementation of key performance mediators. Their Input-Mediator-Output (IMO) model highlights that team performance depends on the quality of inputs ‒ such as role clarity, team composition, and context ‒ as well as on effective communication,^7^ which supports leadership, shared situational awareness,^8^ and other teamwork-related mediators. Haller et al.^9^ demonstrated a direct association between poor communication and adverse events, while Schmutz et al.,^10^ in a meta-analysis, emphasized the relationship between teamwork processes and clinical performance.

The implementation of the WHO Surgical Safety Checklist has contributed to reducing perioperative morbidity and mortality.^11^ As part of this process, team members introduce their names and roles before surgery. Nevertheless, recall of team member identity remains poor, with rates of approximately 30%.^12^^,^^13^ Tools facilitating identification have been shown to optimize team functioning.^14^ The social media initiative #theatercapchallenge, launched in 2018, popularized the use of named caps in operating rooms worldwide. Wearing caps displaying names and professional roles has been associated with improved perceived teamwork and communication,^12^ particularly by facilitating directed communication.^15^ However, no previous study has objectively evaluated the association between named caps and externally assessed team performance. Assessment of team performance remains challenging. One widely used approach relies on validated behavioral assessment tools evaluating non-technical skills.^16^ The TEAM score assesses key domains such as leadership, teamwork, and task management, all of which are known to influence patient safety and quality of care.^17^ Beyond observable behavioral processes, team performance may also be influenced by affective and cognitive dimensions such as group entitativity. Introduced by Campbell,^18^ entitativity refers to the extent to which a collection of individuals is perceived as a coherent and unified group. It can be measured using dedicated scales such as the GEM scale^19^^,^^20^ and includes dimensions such as group cohesion (sense of closeness among group members) and self-inclusion within the group (individual's perceived place within the group).^21^ We hypothesized that named caps could positively influence these perceptions.

The objective of this study was therefore to assess the association between the use of named caps during simulation sessions and team performance, as well as participants’ perceptions of team dynamics, including entitativity, group cohesion, and self-group inclusion.

## Methods

### Study design and population

This was a prospective, single-center, randomized, controlled, and simulation study. The study protocol was approved by the Nimes University Hospital Institutional Review Board (IRB n° 24-12-03).

Inclusion criteria: All medical and paramedical professionals participating in the simulation sessions as part of their continuing professional development were eligible for inclusion. All participants were adults. This included third- and fifth-year anesthesiology and intensive care residents (recently graduated physicians), as well as ICU and operating theatre nurses and nursing assistants. All participants were affiliated with the Montpellier-Nîmes University Hospital (France) and provided written informed consent before participation. Providing consent was mandatory for study participation and constituted the sole exclusion criterion.

**Figure 1:**
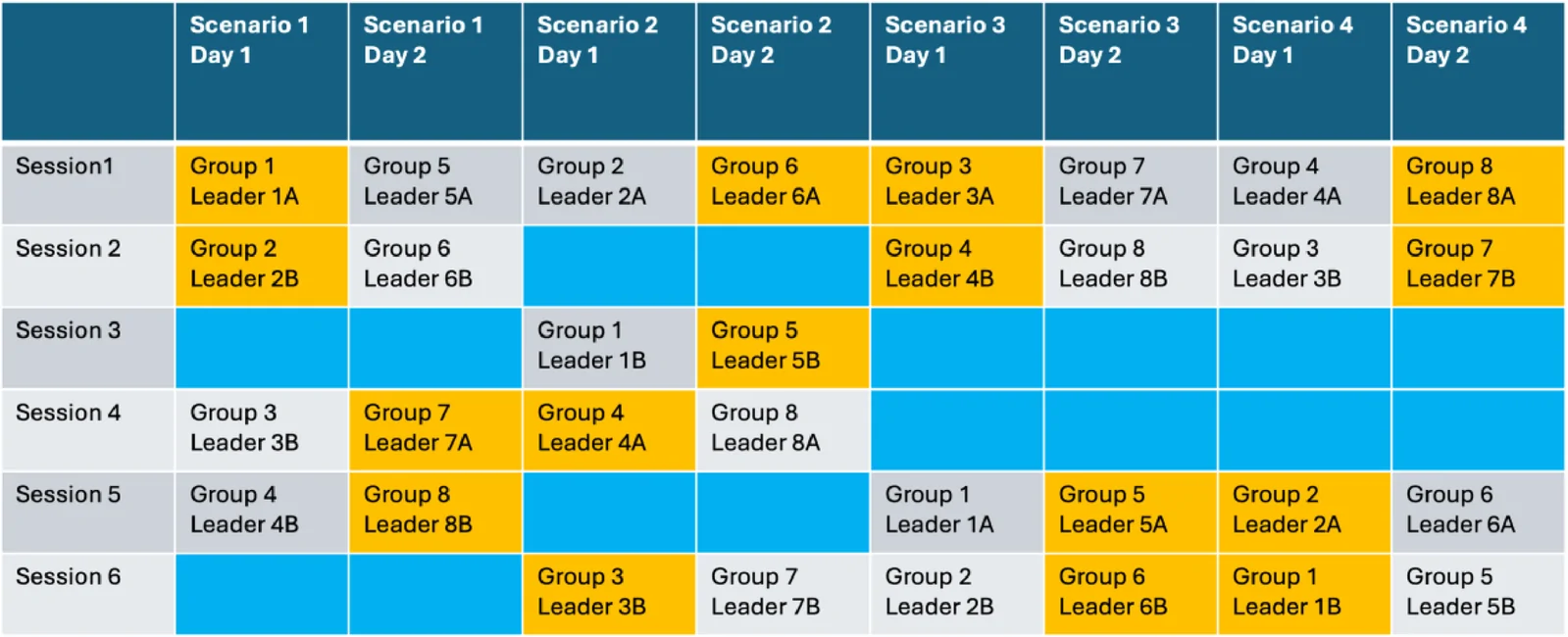
**Session schedule:** Each leader participated in sessions with (orange) or without (grey) named caps. Each scenario was conducted both with and without named caps. Similarly, each team member participated in sessions with (orange) or without (grey) named caps. Sessions 1, 2, and 3 were conducted in the morning, whereas sessions 4, 5, and 6 took place in the afternoon.

Before the simulation sessions, participants attended a general briefing, visited the simulation rooms, and were familiarized with the equipment, particularly the mannequins. Confidentiality, psychological safety, and the right to make mistakes were emphasized. After completion of the scenario and study procedures, participants received a structured debriefing (Fig. 1).

The instructor group consisted of 19 anesthesiologists and healthcare professionals specifically trained in simulation-based medical education and non-technical skills assessment. The simulation scenarios were developed in accordance with the guidelines of the French Society for Simulation in Healthcare (SOFRASIMS) and consisted of standardized high-fidelity scenarios. Four distinct clinical situations were simulated: cardiogenic shock requiring extracorporeal life support, trauma, obstetric anesthesia, and pediatric anesthesia.

Simulation teams consisted of three or four members, including a medical team leader, a resident, a specialized nurse, and, for intensive care scenarios, a nursing assistant. All scenarios had previously been tested at our simulation center (SIMHU, Nîmes University Hospital) using similar team compositions.

### Randomization

Each session was randomized in a 1:1 ratio to either the “named cap” group or the control group. Randomization was stratified by training day and by team. Consequently, each team participated in an equal number of named cap and control sessions. Team composition and scenario order were determined independently and blinded to the randomization process, while the randomization list was prepared by S.G.

Eight groups were constituted, each composed of two leaders and two paramedical teams (one for anesthesia sessions and one for intensive care sessions). Each leader worked with both paramedical teams, and each paramedical team worked with both leaders. Each group participated in four scenarios (Fig. 1).

### Interventions and data collection

The intervention consisted of wearing an identification cap displaying the participant’s name and profession during the named-cap sessions (Material Supplementary Picture 1). In the control sessions, participants either wore plain caps or remained bareheaded. Each team member participated in an equal number of sessions with and without named caps. Sessions without named caps constituted the control group, whereas sessions with named caps constituted the intervention group.

**Figure 2:**
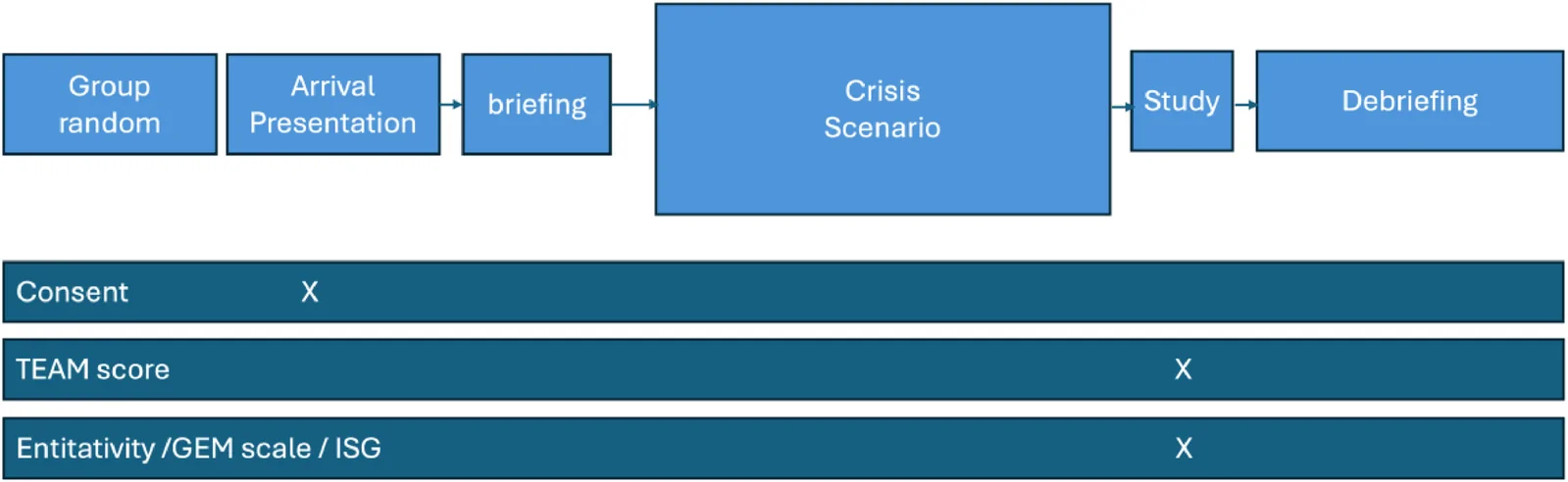
**Study design:** The TEAM score is a validated score for the assessment of non-technical skills.^17^ The GEM scale is a validated scale to evaluate the entitativity of a group.^20^ ISG, Inclusion of Self in the Group.

Data were collected at the end of each session, immediately before the debriefing (Fig. 2). This included TEAM score assessments completed by instructors and team members, as well as participant questionnaires. The TEAM score is a 44 points scale composed of 12 items assessing different domains of non-technical skills, including leadership, teamwork, and task management. In addition, an overall performance 10-point score was recorded.

The observers were not informed of the study objectives. TEAM score assessment was presented as an educational performance evaluation tool intended to support the debriefing process.

### Outcomes

The primary outcome was collective performance, assessed using the TEAM score by simulation instructors, with a focus on non-technical skills.

Secondary outcomes evaluated participants’ perceptions of the team using three self-reported scales: Entitativity, from 0 to 28;^19^ Self-inclusion in the group, using the IOS scale (0–7);^21^ Team cohesion, assessed with an adapted version of the GEM scale (0–7).^20^

### Statistical analysis

No a priori sample size calculation was performed. The sample size was determined pragmatically based on the number of simulation sessions conducted during the study period, and on a previous similar study.^22^ Descriptive statistics were used to summarize participant characteristics, using means and standard deviations or medians and interquartile ranges for continuous variables, and frequencies and percentages for categorical variables. Gaussian distribution of variables was checked graphically. Analyses were performed on complete case basis.

The primary unit of analysis was the simulation session, as TEAM scores were assessed at the session level. We used mixed-effects linear regression models to assess the effect of cap versus control group because the outcome (TEAM score) is continuous and the data have a hierarchical structure, with potential non-independence of observations due to repeated participation of teams across simulation sessions. These models included random intercepts to account for clustering of observations. No additional covariates were included in the model.

Specifically, a random intercept was included for the team, allowing us to account for within-cluster correlation arising from repeated participation across sessions. Instructor-level clustering was not explicitly modeled given the completely anonymous nature of data collection, as no linkage of individuals between sessions could be performed. Model assumptions were assessed by visual inspection of residuals, including Q–Q plots for normality and plots of residuals versus fitted values to evaluate homoscedasticity.

A two-sided p-value < 0.05 was considered statistically significant. All analyses were performed using R software (version 4.0.0; R Core Team, Vienna, Austria).

## Results

In January 2025, a total of 112 participants were included, completing 32 simulation sessions involving 15 different leaders. The 19 instructors provided 58 assessments, with 29 assessments in each group, corresponding to one or two evaluations per session. There were two missing data points in the control group and one in the named cap group.

The simulation sessions were conducted over two days. Eight groups were formed, each comprising two leaders, one ICU team, and one anesthesia team. Each team participated in two sessions led by different leaders ‒ one session with named caps and one without. Similarly, each leader participated in one session with caps (intervention group) and one without caps (control group).

In one group, given only a single leader was available, the same leader conducted all four scenarios, including two sessions with caps and two without.

The questionnaire response rate was 107/112 (95.5%). The characteristics of the study population are detailed in Table 1.

**Table 1:** Characteristics of the study population: ECLS, Extracorporeal Life Support.

Characteristic	Named-cap Group (n = 16)	Control Group (n = 16)
**Scenarios**		
Extracorporeal Life Support (ECLS), n	4	4
Traumatology, n	4	4
Pediatrics, n	4	4
Obstetrics, n	4	4
**Participants**		
Young doctors, n	15	15
Women/Men	7/8	7/8
Third-year residents, n	17	17
Women/Men	9/8	9/8
Nurses, n	16	16
Women/Men	10/6	10/6
Nursing assistants, n	9	9
Women/Men	5/4	5/4
**Completed questionnaires**	52	55
Young doctors, n	16	16
Third-year residents, n	16	16
Nurses, n	16	16
Nursing assistants, n	4	7
**Instructors’ assessments**	29	29

### *Primary outcome: team performance assessment* (Fig. 3)

The TEAM score was significantly higher in the intervention group than in the control group, both for the 44-point TEAM score (37 ± 7 vs. 33 ± 7; p = 0.026) and for the total score (45 ± 8 vs. 40 ± 9; p = 0.020). Detailed results are presented in Table 2.

**Table 2:** Team performance assessment by instructors using the TEAM score.^17^^,^^23^

Assessment	Overall (n = 58)	Control group (n = 29)	Named-cap Group (n = 29)	Mean Difference [95% CI]	p-value
**Leadership**				0.79 [-0.04, 1.6]	p = 0.12
Mean ± SD	6.37 ± 1.60	5.98 ± 1.67	6.77 ± 1.44		
Median (Q1–Q3)	6.0 (5.5–8.0)	6.0 (4.0–8.0)	7.0 (6.0–8.0)		
Range	3.0–8.0	3.0–8.0	3.0–8.0		
Missing data, n	1	0	1		
**Teamwork**				2.6 [0.26, 4.9]	p = 0.031
Mean ± SD	22.5 ± 4.5	21.1 ± 4.4	23.7 ± 4.3		
Median (Q1–Q3)	23.0 (19.5–26.0)	21.0 (17.8–25.6)	24.0 (22.0–27.0)		
Range	11.0–28.0	12.0–28.0	11.0–28.0		
Missing data, n	1	1	0		
**Task Management**				0.64 [-0.12, 1.4]	p = 0.13
Mean ± SD	6.25 ± 1.46	5.93 ± 1.58	6.57 ± 1.28		
Median (Q1–Q3)	7.0 (5.0–7.0)	6.0 (5.0–7.0)	7.0 (6.0–8.0)		
Range	2.0–8.0	2.0–8.0	3.0–8.0		
Missing data, n	0	0	0		
**TEAM Score (/44)**				4.2 [0.60, 7.9]	p = 0.026
Mean ± SD	35 ± 7	33 ± 7	37 ± 7		
Median (Q1–Q3)	35 (31–41)	33 (28–40)	38 (34–43)		
Range	17–44	21–43	17–44		
Missing data, n	2	1	1		
**Overall Performance (/10)**				1.0 [0.16, 1.9]	p = 0.035
Mean ± SD	7.39 ± 1.72	6.86 ± 1.76	7.90 ± 1.54		
Median (Q1–Q3)	8.0 (6.0–8.0)	6.75 (6.0–8.0)	8.0 (8.0–8.0)		
Range	3.0–10.0	3.0–9.5	3.0–10.0		
Missing data, n	1	1	0		
**Total TEAM Score (/54)**				5.4 [0.85, 9.9]	p = 0.020
Mean ± SD	43 ± 9	40 ± 9	45 ± 8		
Median (Q1–Q3)	44 (38–51)	40 (33–48)	46 (42–52)		
Range	20–54	24–52	20–54		
Missing data, n	3	2	1		

**Figure 3:**
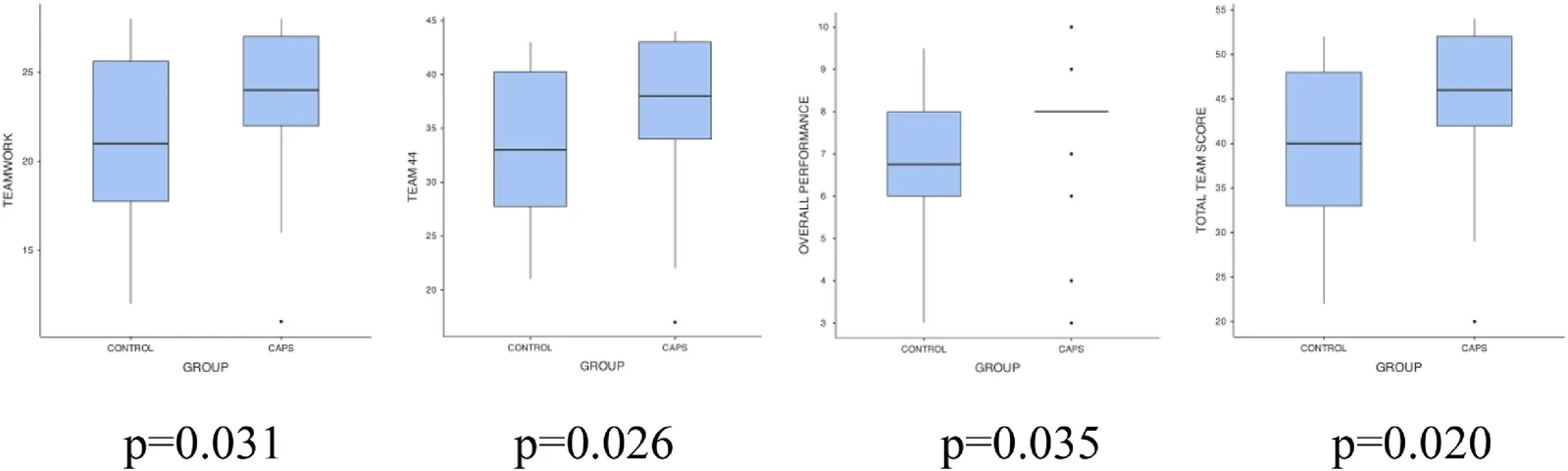
**Comparison for TEAM score between the named-cap and control groups:**^17^^,^^23^ Boxplots show the median, interquartile range (Q1–Q3), minimum, and maximum values. Points represent outliers.

A similar difference was observed for the overall performance rating (7.9 ± 1.54 vs. 6.86 ± 1.76; p = 0.035) and for the teamwork domain (23.7 ± 4.3 vs. 21.1 ± 4.4; p = 0.031) (Fig. 3).

No statistically significant differences were observed in the leadership (p = 0.12) or task management (p = 0.13) domains.

### Secondary outcomes: participants’ perceptions of team functioning

No significant differences were observed between the control and intervention groups regarding perceived entitativity (difference = 0.35 [-1.4; 2.1]; p = 0.8), group cohesion (difference = 0.38 [-0.11; 0.87]; p = 0.4), or self-inclusion in the group (difference = 0.21 [-0.19; 0.61]; p = 0.2) (Table 3).

**Table 3:** Participants’ perceptions of team functioning according to study group: Entitativity, inclusion of self in the group, and group cohesion scores are presented as mean ± standard deviation and median (Q1–Q3).

	Overall (n = 107)	Control Group (n = 55)	Named-cap Group (n = 52)	Mean Difference [95% CI]	p-value
**Entitativity Score**				0.35 [-1.4, 2.1]	p = 0.8
Mean ± SD	22 ± 5	21 ± 4	22 ± 5		
Median (Q1–Q3)	23 (19–25)	22 (18–24)	23 (20–25)		
**Inclusion of Self in the Group (ISG)**				0.21 [-0.19, 0.61]	p = 0.4
Mean ± SD	6 ± 1	6 ± 1	6 ± 1		
Median (Q1–Q3)	6 (5–6)	6 (5–6)	6 (5–6)		
**Group Cohesion (GEM)**				0.38 [-0.11, 0.87]	p = 0.2
Mean ± SD	5 ± 1	5 ± 1	5 ± 1		
Median (Q1–Q3)	5 (4–6)	5 (4–6)	6 (5–6)		

## Discussion

Optimizing teamwork is a major goal for critical care teams and a key mediator of quality of care and patient safety. Healthcare simulation enables teams to be tested and assessed in different contexts. The scenarios used involve critical situations requiring optimal teamwork and are therefore particularly suitable for analyzing team performance. In this study, using named caps was associated with higher team performance, as assessed by the TEAM score, a validated measure of non-technical skills performance.^17^^,^^23^ Although no difference was observed in the leadership domain, significantly higher scores were observed for both teamwork and overall performance. The TEAM score has excellent inter-rater reliability and good construct validity, allowing discrimination between different levels of expertise among simulation teams.^17^^,^^23^^,^^24^ As hypothesized, our findings suggest that named caps may be associated with higher observed team performance in this simulation setting and with the ability of groups to function effectively as teams. Previous studies have suggested an association between wearing named caps and improved communication or self-assessed teamwork efficiency,^12^^,^^25^ and the present study was designed to provide objective and quantitative support for this hypothesis. This represents an important step forward because teamwork assessment tools have only been validated for external evaluation, and limited data are available regarding their use for self-assessment and their validity in this context.^26^^,^^27^ Even though participants introduced themselves and their roles before each simulation session, prior studies have shown that name retention remains poor and that tools facilitating identification can further improve direct communication and role recognition.^4^^,^^5^^,^^12^ Theoretical models of teamwork, particularly the model proposed by Gregory et al.,^6^ emphasize that communication not only directly influences performance but also facilitates the activation of other performance mediators. Improved performance could therefore be expected through enhanced identification and communication. However, the healthcare simulation context ‒ characterized by small groups, general pre-briefing, and individual session debriefing within a psychologically safe environment ‒ already facilitates identification and may optimize performance.^2^^,^^28^ In most cases, each role is assigned to a single individual, and reduced role ambiguity is a well-established factor promoting teamwork and performance, as observed in the sports setting.^29^ Identification may be particularly important in inexperienced, newly formed teams, such as those included in our study, especially in critical situations. The magnitude of the effect observed here, corresponding to an approximately 10% increase in TEAM score, is consistent with previous studies using TEAM,^30^ as well as with simulation studies evaluating the impact of positive communication,^22^ cognitive aids use,^31^ and structured role allocation on team performance during critical situations.^28^

Self-assessment by team members did not reveal any effect of named caps on group entitativity, which was high in both groups, or on its two components: inclusion within the group and group cohesion. The concept of entitativity, introduced by Campbell,^18^ refers to the extent to which a collection of individuals is perceived as a unified entity or a “real” group. Groups with high entitativity are considered as having greater capacity for collective action.^32^ Key drivers of entitativity include a shared goal, interdependence, effective communication, and shared values such as benevolence and security,^33^ all of which are fostered by the healthcare simulation environment. Conversely, factors such as the duration of group membership and the intensity of shared group experience also enhance group identity. These were absent in our transient teams, which had no previous or future collective experience. Entitativity is linked to two dimensions in social psychology: group cohesion and individual self-inclusion within the group. These two dimensions were assessed separately in our study and were high in both groups, with no significant differences. Although this separation remains debatable, as these metrics are traditionally assessed together, our findings suggest that respondents meaningfully differentiated between these two dimensions, supporting the validity of this analytical approach. Assessing these dimensions in clinical settings within established care teams could, therefore, be of interest.

Self-inclusion reflects individuals’ perceived position within the group. In our study, participants reported a high level of integration, although the study design and limited sample size did not allow further analyses according to professional category. Nevertheless, the higher performance observed in teams wearing named caps was not associated with different perceptions among team members, reinforcing the hypothesis that named caps may improve non-technical skills rather than reflecting differences in team commitment.

Moreover, the implementation of performance mediators, as measured by the TEAM score, may lead to performance adaptation through affective, behavioral, and cognitive mechanisms. In this study, we hypothesized that the intervention would promote affective effects, including a sense of cohesion, entitativity, and inclusion within the team, but it was unable to demonstrate such effects. However, the simulation context, particularly repeated exposure to critical situations, may have reinforced these feelings in both groups, resulting in a ceiling effect. Another explanation is that performance may involve other affective mechanisms not assessed here, such as stress, as well as behavioral and cognitive mechanisms. For example, named caps promote the use of first names,^4^ which may help capture attention.^34^^,^^35^ Finally, implicit processes, such as inter-personal synchrony or co-representation, may also be involved,^36^^,^^37^ but could not be assessed in this study.

The main limitation of our study was that participants could not be fully blinded to the intervention, as wearing named caps inherently revealed the study objective. Although all instructors were qualified assessors, and their primary role was educational, the presence of caps may have influenced their perception, leading to observer bias. Observer-based assessments of team performance remain subjective and prone to bias and noise.^38^ This raises broader questions regarding the evaluation of team performance by external observers, particularly when the intervention is highly visible, as in the present study. Automated systems, such as those already integrated into operating room “black boxes” and using artificial intelligence, may help standardize and streamline such evaluations in the future.^39^ Experimental studies also suggest that analysis of inter-individual synchrony, including inter-brain, behavioral, and physiological synchrony, could contribute to the assessment of team performance.^37^ Team performance assessment remains a major challenge for understanding the determinants of performance and helping teams improve, particularly through simulation training. The teams studied were formed specifically for the simulation training session. Therefore, it was not possible to measure the prior level of knowledge within each team. Similarly, prior team experience with the clinical situations presented in the scenarios was not assessed. These factors may represent potential confounders. However, since all leaders were junior physicians with no prior experience acting as senior physicians within those paramedical teams, and since they had the same level of academic training, the impact of these confounders may have been limited. The simulated setting also ensured optimal support and psychological safety. Therefore, our results are valid only within such an environment, which specifically allows the use of first names in communication. Named caps should be considered as part of a broader human factor strategy.^40^ In this study, cap tolerance was not specifically assessed. However, no discomfort or adverse effects were reported.

The effect of named caps may also have been enhanced by the fact that teams were newly formed and leaders relatively inexperienced. Their impact should therefore be assessed in other circumstances, particularly among experienced teams and in ecological clinical settings. Conversely, the teams studied here were small and composed of members with clearly defined roles. Future studies could test this intervention in larger teams with higher turnover.

We also could not account for clustering at the instructor level, which may have introduced residual dependence in our analyses. Finally, the absence of an a priori sample size calculation is a limitation, as the study may have been underpowered to detect small but clinically relevant differences.

Overall, these findings highlight the need for larger-scale evaluation under real-life conditions, such as in operating rooms and/or intensive care units, to confirm the impact of improved identification on team performance in both critical and routine situations, and to determine whether this intervention can ultimately improve patient outcomes.

## Conclusion

Facilitated identification of team members using named caps was associated with higher observed team performance in this simulation study. No statistically significant effect was observed on perceived group cohesion, self-inclusion, or group entitativity. These findings should be validated in larger real-life clinical settings before conclusions can be drawn regarding patient outcomes.

## Data availability statement

The datasets generated and/or analyzed during the current study are available from the corresponding author upon reasonable request.

## AI assistance disclosure

The authors used ChatGPT (OpenAI) to improve the English language and readability of the manuscript. The authors reviewed and edited the output and take full responsibility for the content.

## Study registry

Not applicable – Not a clinical study.

## Authors’ contributions

Study design: SG, LB, CR, GB. Recruitment of participants: SG, CR, LMa. Randomization: SG. Data acquisition: SG, CR, LMa. Interpretation of results: SG, CR, CS. Statistical analyses: CS. Writing of the first version of the article: SG, CR, LMu. Critical revision of the article for intellectual content: GB, LB, PC, NB, SB. Final approval of the version to be published and acceptance of responsibility for all aspects of the work, thus ensuring that questions related to the accuracy or integrity of any part of the work are properly investigated and resolved: All authors.

## Conflicts of interest

The authors declare no conflicts of interest.

## Appendix Supplementary materials

Image, application 1

## Footnotes

Supplementary material associated with this article can be found in the online version at doi:10.1016/j.bjane.2026.844812.
