# Supplementary material for: Impact of named caps on anesthesia and intensive care team performance: a randomized simulation study
Source: Braz J Anesthesiol. 2026 Sep 4;76(5):844812. doi: 10.1016/j.bjane.2026.844812 (PMC13572307; doi:10.1016/j.bjane.2026.844812)
Supplement: Supplementary file 1 [file mmc1.docx]

**BJAN-D-25-00506_Supplementary Material**

**Supplementary Material** Photography.

**Figure 1** Intensive care team wearing named caps during a simulation session.
